# Rupture Uterus in a Tertiary Care Centre: A Descriptive Cross-sectional Study

**DOI:** 10.31729/jnma.5894

**Published:** 2021-04-30

**Authors:** Deepa Chudal, Sebak Shrestha, Ruby Shrestha, Vikash Paudel

**Affiliations:** 1 Nepal Police Hospital, Kathmandu, Nepal; 2 National Medical College, Birgunj, Kathmandu, Nepal

**Keywords:** *caesarean section*, *maternal mortality*, *perinatal mortality*, *rupture uterus*

## Abstract

**Introduction:**

Rupture uterus is an obstetric catastrophe with poor maternal and fetal outcome. The objective of the study is to determine the prevalence of rupture uterus in pregnancy.

**Methods:**

This was a descriptive cross sectional study conducted in a tertiary care centre from January 2016 to December 2016 after taking ethical approval (Approval No. F-NMC-510/ 76/ 77) from Institutional Review Committee. Convenience sampling method was used. Data were entered in the Microsoft Excel sheet and obtained data was analysed using Statistical Package for Social Sciences version 18 software for central tendency and frequencies.

**Results:**

Out of total 1559 deliveries, prevalence of rupture uterus was found to be 12 (0.77%). Previous lower segment caesarean scar rupture was the most common risk factor noted in 7 (58.3%) cases. A total of seven patients (58.3%) required intensive care unit admission and blood transfusion. Other maternal complications were surgical site infection 2 (16.67%), sepsis 2 (16.67%), paralytic ileus 1 (8.3%), pelvic collection 1 (8.3%) and vesico vaginal fistula 1 (8.3%). Two maternal deaths (16.67%) and perinatal death was noted in 8 (66.66 % ) cases.

**Conclusions:**

Rupture uterus most commonly occurred in scarred uterus. Identification of high risk pregnancy, judicious caesarean section, proper labor monitoring, early diagnosis and prompt management are essential in reducing its occurrences.

## INTRODUCTION

Rupture of a gravid uterus refers to tear of the uterine muscle occurring during pregnancy, delivery, or immediately after delivery. ^1^ Various factors associated with increased risk of uterine rupture include previous cesarean section, uterine scars, uterine anomalies, grand multiparty, use of oxytocin, placenta percreta, low socioeconomic class, prolonged obstructed labor and delayed management of labor. ^2,3^

Uterine rupture is more prevalent in less developed countries like Nepal.^4^ It's a life-threatening emergency resulting in maternal death (0-1% in modern developed nations and 5-10% in developing countries). ^5^ Here, primary rupture with more disastrous complications is common than scar rupture. However, the etiological trend is changing due to a rise in caesarean deliveries leading to uterine scars and future risk of rupture. ^6^

This study was conducted to determine the prevalence of ruptured uterus.

## METHODS

This was a descriptive cross sectional study conducted in department of Obstetrics and Gynecology in National Medical College and Teaching Hospital, a tertiary level referral center in Central Terai region of Nepal with high number of obstetric cases. The duration of the study was one year from January 2016 to December 2016. Ethical approval was taken from the Institutional Review Committee (Approval No.: F-NMC-510/76/77). All cases of complete and incomplete uterine rupture including scar dehiscence. Whole sampling method was used and minimum sample size was calculated using formula:

$$\begin{array}{ll} \text{n} & \text{= }{\text{Z}}^{\text{2}}\times (\text{p}\times \text{q)}/{\text{e}}^{\text{2}}\\ & \text{= }1.96^{\text{2}}\times (0.5\times 0.5)/\text{0}{\text{.03}}^{\text{2}}\\ & \text{= }1068 \end{array}$$

where,

n = sample size
p = prevalence of rupture uterus, 50%
q = 1-p
e = margin of error, 3%
Z = 1.96 at 95% CI

The minimum sample size calculated was 1068 and sample of 1559 was taken. A preformed proforma was designed by investigators to record all the available demographic details and clinical parameters. Identification of all cases was done through the departmental obstetrics register before the case note files were retrieved. Case record file with diagnosis of rupture uterus were retrieved from the records department and information on maternal demographic characteristics, risk factors, induction or augmentation of labour, medical or surgical intervention, instrumentation, intrauterine manipulation, maternal and perinatal outcome were recorded using a proforma. Perinatal and maternal outcomes of the cases were also reviewed. Every relevant information was entered in the Microsoft Excel sheet and obtained data was analysed using Satistical Package for Social Sciences version 18 software for central tendency and frequencies.

## RESULTS

During the study period of one year from January 2016 to December 2016, out of total 1559 deliveries, prevalence of rupture uterus was found to be 12 (0.77%). The age range of patients was from 20 years to 35 years out of which 7 (58.3%) were of 20-25 years. Out of 12 cases, 10 (83.3%) were referred cases and only 2 (16.6%) were booked case of our hospital. The mean parity and gestational age were 2 and 38.4 weeks respectively. Uterine rupture was noted in term pregnancies between 37 weeks and 42 weeks in 11(91.6% ) cases and one preterm rupture occurred at 36 weeks' gestation in the previously scarred uterus (Table 1).

**Table 1:** Demographic and clinical characteristics (n=12).

Characteristics	n (%)
Age in years	
20-25	7 (58.3)
26-30	3 (25)
>30	2 (16.67)
Parity	
P1	8 (66.67)
P2-4	2 (16.67)
P>-5	2 (16.67)
Booking status	
Booked	2 (16.67)
Referred	1O (83.3)
Gestational age in weeks	
<37	1 (8.3)
37-42	11 (91.66)
>42	0(0)

Rupture of the previous lower segment caesarean section scar was noted in 7 (58.3%) cases. Complete uterine rupture was noted in 8(66.67%) cases with the lower uterine segment being the common site of rupture. All cases of rupture occurred during the intrapartum period after the onset of labour. Repair of the rupture site with bilateral tubal ligation was done in 8 (66.67%) cases followed by subtotal hysterectomy in the remaining cases (Table 2).

**Table 2:** Risk factors, characteristics and management of uterine rupture

Characteristics	n (%)
Risk factors	
Previous scar	7 (58.3)
Obstructed labour	2 (16.67)
Instrumental delivery	2 (16.67)
Mal-presentation	1 (8.3)
Type of rupture	
Complete	8 (66.67)
Incomplete	4 (33.3)
Site of rupture	
1) Lower uterine segment	
Anterior wall with or without extension into broad ligament	7 (58.3)
Posterior wall	1 (8.3)
2) Upper uterine segment	
Posterior wall	2 (16.67)
Anterior wall	1 (8.3)
Right lateral wall	1 (8.3)
Time of rupture	
Antepartum	0(0)
Intrapartum	12 (100)
Surgical management	
Repair with sterilization	8 (66.67)
Obstetric hysterectomy	4 (33.3)

Various maternal complications following uterine rupture is presented in (Table 3). Seven (58.3%) cases required intensive care unit admission and blood transfusion. There were 2 (16.6%) cases each of surgical site infection and sepsis. There were 2(16.67%) maternal deaths during the study period due hypovolemic shock. Perinatal mortality was seen in 8 (66.67%) cases; among them seven were fresh still birth and one was early neonatal death (Table 4).

**Table 3:** Maternal morbidity and mortality following ruptured uterus.

Variable	n (%)
Obstetric haemorrhage requiring blood transfusion	7 (58.3)
Need of ICU admission	7 (58.3)
Surgical site infection	2 (16.67)
Sepsis	2(16.67)
Paralytic ileus	1 (8.3)
Pelvic collection	1 (8.3)
Vesico-vaginal fistula	1 (8.3)
Prolonged hospital stay > 7 days	7 (58.3)
Mortality	2 (16.67)

**Table 4:** Perinatal outcome following uterine rupture.

Variable	n (%)
Live birth	4 (33.3)
Fresh Stillbirth	7 (58.3)
Early neonatal death	1 (8.3)
Perinatal mortality	8 (66.67)

## DISCUSSION

Rupture of the gravid uterus leads to grave complications endangering the life of both the mother and baby. Despite its rarity, it still contributes to significant maternal and perinatal morbidity and mortality especially in the setting of developing countries like Nepal.

The prevalence of rupture uterus was 0.76% in our study which is greater than 0.071% in the study done at a tertiary centre in Kathmandu.^7^ Another study in eastern Nepal showed the prevalence of rupture to be 0.45%.^5^ The increased prevalence of rupture in our study may be due to the delay in access to the tertiary level hospital owing to poverty, illiteracy, lack of family planning and women empowerment especially in rural Terai region of Nepal. Many high-risk cases for rupture like previous caesarean section, grand-multipara women are given a trial of labor at home by untrained dais even with oxytocics injudiciously with no proper diagnosis of rupture and there is delayed referral with most women landing up in severe shock or moribund situation. The prevalence of rupture uterus was 0.9% in a cross-sectional study conducted in a tertiary care hospital of Ethiopia reflecting even more worse conditions than our setting. ^8^

Ten (83.3%) cases presented between the ages of 20-30 years and the mean parity was two. Uterine rupture occurred in 11 (91.6%) in gravida 2-4 with only one patient being grand multipara. The age and parity distribution of our study were concomitant with the findings of other studies.^9,10^

Unbooked status of the patient was found to be one of the significant factors associated with rupture uterus in a Nigerian study which was similar to the finding of our study in which 83.3% cases were unbooked.^3^ This reflects the substantial ignorance in the management of high risk cases and poor access to the tertiary health care center.

In our study, rupture of the previous caesarean scar accounted for 58.3% of rupture cases followed by obstructed labor 16.6%, instrumental delivery 16.6% and malpresentation 8.3%. Other studies conducted in different sites in Nepal also showed previous caesarean scar rupture to be the most common cause of rupture with percentage being much higher (72-78%) than our study.^5,7,11^ Sunanda N, et al. also concluded that separation of previous caesarean section scar was the commonest cause of rupture in their two-year analysis of uterine rupture in pregnancy in Mysore, India.^12^ This is due to the increasing trend of caesarean section in modern obstetrics. Lack of counseling for contraception, short inter-delivery interval, poor acknowledgement of the scarred uterus as a major risk factor, delay in diagnosis of rupture and delayed referral often contributes to scar rupture. Management of scarred uterus by skilled manpower in an appropriately equipped health care facilities with meticulous supervision for cases undergoing a trial of labor is strongly advocated to reduce this disastrous complication.

Even when there is suspicion of uterine rupture, prompt surgical intervention should be taken to avoid the dreadful consequences of severe maternal and perinatal morbidity and mortality. The choice between repair of rupture or hysterectomy depends upon the type, site, extent of rupture as well as the clinical condition of the patient. Repair of the the rupture site with bilateral tubal ligation was done in 8 (66.66%) cases, while subtotal hysterectomy was performed in 4 (33.33%) cases. Similarly, repair of rupture site was considered main and safer modality of treatment in other studies.^9,13^ This might have the advantage of maintaining reproductive capability and menstruation but with increased risk of recurrent rupture uterus in subsequent pregnancy.^14^ Life threatening obstetric hemorrhage can occur following uterine rupture with need of blood transfusion which was done in 58.3% cases in our study. Requirement of blood transfusion in most of the cases of uterine rupture has been noted in other studies as well.^11,14,15^ These results emphasizes the utmost importance of effective blood banking services and availability of blood and blood products especially during the peri-operative period for better outcome of the patient.

Surgical site infection 16.67%, sepsis 16.67%, pelvic collection 8.3% and paralytic ileus 8.3% were the complications following rupture in our study. One patient developed vesicovaginal fistula. Ahmed et al. found similar morbidities like rectovaginal fistula, ICU admission, wound dehiscence and sepsis in their study. ^8^ As per WHO systematic review 2005, maternal mortality between 1 and 13% and perinatal mortality between 74% to 92% have been reported following uterine rupture. ^4^ There were two maternal deaths 16.67% with a perinatal mortality of 66.66% in our study. Similar observation with maternal mortality 16% and perinatal mortality of 72.9% was noted in a study conducted in Mumbai by Ganesh et al.^16^ But there were studies where they had no maternal mortality in their retrospective study.^14^ Delayed referral and late arrival in hospital in the state of hypovolemic shock was the main reason for high maternal and perinatal mortality in our study. The limitation of this study were its retrospective design, small samle size and single institution study.

## CONCLUSIONS

Uterine rupture, often a preventable condition is a serious complication and a major contributor to maternal morbidity and neonatal mortality. Previous caesarean scar rupture is the most common cause of rupture. Promotion of skilled birth attendant, identification of high risk pregnancy, judicious caesarean section, cautious use of oxytocic drugs, proper labor monitoring and education about supervised pregnancy and institutional delivery are essential in reducing its occurrence.

## References

[1] Manoharan M, Wuntakal R, Erskine K (2010). Uterine rupture: a revisit. The Obstetrician & Gynaecologist.

[2] Ofir K, Sheiner E, Levy A, Katz M, Mazor M (2003). Uterine rupture: risk factors and pregnancy outcome. Am J Obstet Gynecol.

[3] Al-Zirqi I, Daltveit AK, Forsen L, Stray-Pedersen B, Vangen S (2017). Risk factors for complete uterine rupture. Am J Obstet Gynecol.

[4] Hofmeyr GJ, Say L, Gulmezoglu AM (2005). WHO systematic review of maternal mortality and morbidity: the prevalence of uterine rupture. BJOG.

[5] Gerard G, Nahum M, FACS. (2021). Uterine Rupture in Pregnancy [Internet].

[6] Pokhrel Ghimire S (2018). Uterine Rupture: Shifting Paradigm in Etiology. Kathmandu Univ Med J.

[7] Uprety I, Baral G, Shrestha S (2018). Review of uterine rupture at Paropakar Maternity and Women's Hospital. NJOG.

[8] Ahmed DM, Mengistu TS, Endalamaw AG (2018). Incidence and factors associated with outcomes of uterine rupture among women delivered at Felegehiwot referral hospital, Bahir Dar, Ethiopia: cross sectional study. BMC Pregnancy Childbirth.

[9] Aziz N, Yousfani S (2015). Analysis of uterine rupture at university teaching hospital Pakistan. Pak J Med Sci.

[10] Sahu M, Natasha HK, Mandpe P (2016). Case analysis of complete uterine rupture in a tertiary health care center. Int J Reprod Contracept Obstet Gynecol.

[11] Shrestha J, Shrestha R (2015). Analyzing uterine rupture: A study from tertiary care centre of western Nepal. Journal of Kathmandu Medical College.

[12] Sunanda N, Priya R (2016). A two-year analysis of uterine rupture in pregnancy. Int J Reprod Contracept Obstet Gynecol.

[13] ACOG Practice bulletin no. 115: Vaginal birth after previous cesarean delivery. (2010). Obstet Gynecol.

[14] Sinha M, Gupta R, Gupta P, Rani R, Kaur R, Singh R (2016). Uterine Rupture: A Seven Year Review at a Tertiary Care Hospital in New Delhi, India. Indian J Community Med.

[15] Mistry S, Sarmalkar M, Nayak AA (2017). Study of maternal outcome in uterine rupture in pregnancy at a Tertiary Care Institute. IJBAR.

[16] Shinde G, Pawar A, Jaisal P, Jadhav B (2011). Maternal and perinatal outcome of rupture uterus at tertiary care centre. Bombay Hospital Journal.

